# Ligature of Both Carotid Arteries for a Remarkable Erectile Tumour of the Mouth, Face and Neck

**Published:** 1846-06-01

**Authors:** 


*Ligature of both Carotid Arteries for a Remarkable Erectile Tumour of the Mouth, Face and Neck, by* J. Mason Warren, M. D.—American Journal of Medical Science, for April, 1846.

A ligature was first applied to the carotid artery forty years ago, by Sir Astley Cooper. To Dr. Mussey is due the credit of first ligating both carotids in the same subject in this country, and, if we mistake not, he was not anticipated abroad. This was in 1827. The operation has since been repeated in several instances. Its safety and practicability are therefore established. "The great object," Dr. Warren states to be, "that sufficient time should elapse between the ligatures of the carotids to allow the collateral vessels which supply the brain to be dilated so as to carry the quantity of blood required for the performance of its functions." In the case reported by Dr. Warren, the effect upon the tumour was salutary, and the success of the operation as great as could reasonably have been anticipated. Four months after its performance, the patient was in the enjoyment of perfect health, not having had the slightest indications of disturbance in the brain from the interruption of its natural circulation.

These cases are not without interest in a pathological point of view. With our present views of cerebral congestion as dependent on anæmia, either from the fact of there being too little blood in the vessels, or from a want of power in the forces carrying on the circulation, should we not expect that interrupting the flow through both carotids, would be followed, at least, by the same pathological condition? Does not the fact that it is otherwise go to show that that the pathology of anæmia, in so far as the brain is concerned, has more to do with the quality of the blood than the quantity sent to the brain? Do not the cases of this operation also show that the rationale of syncope usually received requires some modifications?

